# MARCH6 promotes Papillary Thyroid Cancer development by destabilizing DHX9

**DOI:** 10.7150/ijbs.60628

**Published:** 2021-08-03

**Authors:** Yang Liu, Siyuan Xu, Ying Huang, Shaoyan Liu, Zhengang Xu, Minghui Wei, Jie Liu

**Affiliations:** 1 Department of Head and Neck Surgical Oncology, National Cancer Center/National Clinical Research Center for Cancer/Cancer Hospital, Chinese Academy of Medical Sciences and Peking Union Medical College, Beijing, P. R. China.; 2 Department of Head and Neck Surgical Oncology, National Cancer Center/National Clinical Research Center for Cancer/Cancer Hospital & Shenzhen Hospital, Chinese Academy of Medical Sciences and Peking Union Medical College, Shenzhen, P. R. China.

**Keywords:** Papillary thyroid cancer, MARCH6, Tumorigenesis, DHX9, AKT/mTOR

## Abstract

Membrane-associated ring-CH-type finger (MARCH) proteins belong to the E3 ubiquitin ligase family, which regulates protein stability by increasing ubiquitination. Recent evidence has shown that some MARCH proteins play important roles in cancer development. However, the role of MARCH6 in tumorigenesis, including thyroid tumorigenesis, remains unknown. In this study, we determined that MARCH6 was upregulated in the majority of primary papillary thyroid cancers (PTCs) at both the mRNA and protein levels. Gain-of-function and loss-of-function studies demonstrated that MARCH6 suppressed apoptosis and promoted cell cycle progression, cell proliferation, growth, migration and tumorigenesis in thyroid cancer cells. Mechanistically, MARCH6 interacted with and downregulated DHX9. Knockdown of DHX9 enhanced the proliferative and migratory abilities of thyroid cancer cells. The inhibitory effect of MARCH6 knockdown on thyroid cancer cell growth and migration was also reversed by DHX9 silencing. In addition, MARCH6 activated the AKT/mTOR signaling pathway in a manner dependent on the downregulation of DHX9. Overall, MARCH6 functions as a potential oncogene in thyroid cancer by destabilizing DHX9 and activating AKT/mTOR signaling.

## Introduction

Thyroid cancer (TC) is one of the most common endocrine malignancies, with a rapidly increasing incidence worldwide, which has been referred to as an epidemic of overdiagnosis 1. According to epidemiological studies, the incidence rate of thyroid cancer has increased 240% over the past decades 2. Moreover, 4 histological types of thyroid cancers have been reported, namely, papillary, follicular, medullary and poorly differentiated, which reflects the significant heterogeneity of thyroid cancer. Importantly, this heterogeneity consists of not only histopathological diversity but also differences in multiple genetic and epigenetic alterations, resulting in greater challenges for the diagnosis and prognosis of high-risk thyroid cancer patients 3. In recent years, numerous efforts have been made to identify appropriate molecular markers to represent thyroid cancer heterogeneity 4, 5. Nevertheless, novel molecular biomarkers are still needed to predict the malignancy of thyroid tumors.

Since protein ubiquitination, one of the most common posttranslational modifications, is regarded as a natural mechanism involved in the occurrence and development of cancers, dysregulation of its associated pathways could contribute to abnormal cell proliferation and invasion processes, ultimately resulting in the development of gland tumors 6, 7. Among the ubiquitin-associated molecules, the family of membrane-associated ring-CH-type finger (MARCH) proteins has been reported to regulate protein stability and other properties of substrates by increasing ubiquitination. MARCH6, a member of the MARCH family also known as TEB4 or RNF176, is an E3 ubiquitin ligase localized to the endoplasmic reticulum 8. Increasing studies have suggested that MARCH6 plays important roles in multiple tumors either by regulating posttranscriptional modifications to affect enzyme substrates or by protein-protein interactions 9-13. However, the specific functions of MARCH6 in thyroid cancer progression are still unidentified.

DExH-box RNA helicase 9 (DHX9), also named RHA or NDHII, belongs to the EDAH RNA helicase family and possesses DNA-RNA helicase activity, which is necessary for the efficient resolution of DNA damage repair-induced R-loops important for double-strand break (DSB) repair 14. DHX9 was initially found to act as a nucleic acid sensor in antiviral immunity 15. A recently, study indicated that DHX9 suppresses the EMT process in human lung cancer cells by targeting STAT 16. Additionally, DHX9 interacts with NONO and SFPQ to form a DHX9-NONO-SFPQ complex, a key regulator in liver cancer 17. However, whether DHX9 plays important roles in thyroid cancer development is still unclear.

In the present study, we aimed to investigate the functional roles of MARCH6 in the progression of thyroid cancer. We found that MARCH6 is upregulated in primary papillary thyroid cancers (PTCs), and this upregulation contributes to the promotion of thyroid cancer cell proliferation, migration and cell cycle progression. Furthermore, we determined that MARCH6 could interact with DHX9 to regulate thyroid cancer cell growth and migration. In addition, MARCH6 also activated the AKT/mTOR signaling pathway and downregulated DHX9.

## Methods and Materials

### Cell culture

The thyroid cancer cell lines BCPAP, K1 and TPC-1 were purchased from ATCC. BCPAP, K1 and TPC-1 cells were cultured with DMEM-HG or RPMI-1640 (Gibco) medium supplemented with 100 U/mL penicillin, 5 mg/mL streptomycin and 10% fetal bovine serum (FBS, Gibco). All the cells were maintained in an incubator at 37 °C with a humidified 5% CO_2_ atmosphere.

### Patients and specimens

All clinical tissue samples from patients with PTC and normal adjacent tissues were obtained from populations in China and were collected at the Cancer Hospital, Chinese Academy of Medical Sciences and Peking Union Medical College between 2016 and 2019. The research was approved by the Ethics Committee of National Cancer Center/National Clinical Research Center for Cancer/Cancer Hospital, Chinese Academy of Medical Sciences and Peking Union Medical College and conducted under the guidance of the Declaration of Helsinki. Informed consent regarding the use of specimens was obtained from all patients. All clinical samples were histopathologically diagnosed by 2-3 pathologists.

### Immunohistochemical staining of MARCH6

IHC staining of MARCH6 on 4-5 um of paraffin-embedded tissue section was conducted according to the protocols as described previously 18. In brief, the tissue section was subjected to deparaffinization, antigen retrieval, blockage of endogenous peroxidase, antibody incubation (1:200) and DAB (3,3′-Diaminobenzidine) coloration.

### Lentivirus-mediated MARCH6 knockdown and overexpression

The lentivirus vector system consisting of the pGCSIL-GFP, pHelper1.0 and pHelper2.0 vectors was applied for MARCH6 knockdown and overexpression in thyroid cancer cell lines. The sequences of the MARCH6 and DHX9 shRNAs were as follows: shMARCH6#1: 5'-GCACACTGTGTGCATTCATCA-3'; shMARCH6#2: 5'-GCTTACTGGGAGTCTGCTATA-3'; and shDHX9: 5'-GGGCTATATCCATCGAAATTT-3'. After cloning the shRNAs into the pGCSIL-GFP vector, each corresponding vector was cotransfected with pHelper1.0 and Helper2.0 into 293T cells, after which the viral supernatants were harvested by centrifugation and stored at -80°C until use. The knockdown and overexpression efficiencies were determined by qRT-PCR and western blot assays.

### CCK-8 assay

A Cell Counting Kit-8 (CCK-8) assay was performed to determine the cell proliferation capacity. Briefly, 1.0×104 BCPAP, K1 and TPC-1 cells transduced with shCtrl, shMARCH6, Ctrl or MARCH6-OE were separately seeded into 96-well plates and maintained at 37 °C for 5 days. After cell culture for 1, 2, 3 and 4 days, 20 μl CCK-8 reagent was added to each well, and cell culture was continued for another 3 h. Then, cell growth curves were generated according to the optimal density, which was measured at 450 nm by a microplate reader.

### Colony formation assay

Equal numbers of shCtrl-, shMARCH6-, Ctrl- and MARCH6-OE-treated BCPAP, K1 and TPC-1 cells were separately seeded into 6-well plates and cultured with complete medium for 10 days. During this period, the medium was replaced every 3 days. Then, the cells were fixed with methanol and stained with Giemsa solution (Beyotime, China). The colony numbers were counted, and images were taken using a camera.

### Transwell assay

Transwell assays were used to determine the cell migration ability. For the Transwell (24-well, 8-mm pore size, Corning Costar, USA) assay, 1.0×10^5^ BCPAP, K1 and TPC-1 cells were seeded on the uncoated membrane of the upper chamber and maintained in medium without serum. Then, 0.6 mL medium supplemented with 10% FBS was added into the lower chamber of the Transwell. After incubation for 20 h, the nonmigrating cells in the upper chamber were removed with a cotton swab, and the migrating cells were stained with crystal violet (Beyotime, China). The numbers of migrating cells were counted from at least 5 random fields, and then Transwell images were taken.

### Cell cycle assay

The cell cycle profile was analyzed with propidium iodide (PI) staining as previously described. In brief, 1.0×10^6^ BCPAP, K1 and TPC-1 cells were washed twice with PBS, treated with trypsin and centrifuged to harvest the cell pellet. After fixation with cold 70% ethanol at 4 °C for 30 min, the cell pellet was incubated with a solution containing 5 μl Annexin V-FITC and 1 μl PI (50 μg/mL). Then, flow cytometry was conducted to determine the proportion of cells in each phase of the cell cycle, and the data were analyzed using a FACScan (BD, USA). The experiment was repeated at least three times.

### Cell apoptosis assay

To assay cell apoptosis, stably transduced BCPAP, K1 and TPC-1 cells from the different groups (shCtrl, shMARCH6, Ctrl and MARCH6-OE) were cultured for 24 h, fixed with ethanol and then stained with PI as previously described. Then, the cells were analyzed by flow cytometry. The experiments were performed at least three independent times.

### *In vivo* xenograft study

TPC-1 cells from different groups (shCtrl, shMARCH6, Ctrl and MARCH6-OE) were subcutaneously injected into the right armpits of 4-6-week-old female Balb/c nude mice. Each group contained five mice. The tumor volume was calculated every three days after cell implantation, according to the formula *V*=*ab*^2^/2, where *a* is the long diameter and *b* is the short diameter. After implantation for 35 (for Ctrl and MARCH6 overexpression group) or 45 days (for shCtrl and shMARCH6 group), the mice were sacrificed, and the tumors were removed from the mice for further investigation.

### qRT-PCR assay

To quantify gene expression at the mRNA level, cells were seeded into 24-well plates. After specified treatments for 48 h, total RNA was harvested using TRIzol (Invitrogen) reagent, and the Reverse Transcription System Kit (Promega) was applied for reverse transcription of RNA into cDNA. qPCR was conducted using a StepOne system with SYBR Master Mix (TaKaRa). The mRNA expression levels of each target gene were normalized to those of the housekeeping gene beta actin. The sequences of the primers were as follows: MARCH6-F: 5'-ACCAGAGGAGTTCTTGACCCCAAA-3'; MARCH6-R: 5'-CCCCAGAATCACCCGAGCAG-3'; beta actin-F: 5'-GGCTGTATTCCCCTCCATCG-3'; beta actin-R: 5'-CCAGTTGGTAACAATGCCATGT-3'.

### Western blot assay

Total protein lysates of thyroid cancer cells and tissues were obtained using RIPA lysis buffer (Beyotime, China) supplemented with 1 mM proteinase inhibitor cocktail (Roche, Germany). Thirty micrograms of immunoprecipitated proteins was resolved and immunoblotted by SDS-PAGE as previously performed by using antibodies against the following antigens: MARCH6 (CST), DHX9 (CST), p-mTOR (CST), mTOR (CST), p-AKT (CST), AKT (CST), p-S6 (CST), S6 (CST), beta actin (CST) and GAPDH (CST). Beta actin or GAPDH was used as an endogenous control. Peroxidase-conjugated anti-mouse or anti-rabbit IgG (CST) was used as the secondary antibody, and the western blot results were visualized by the ECL assay (Millipore, USA).

### Statistical analysis

All data are presented as the mean ± standard error of the mean. SPSS software was used for the statistical analysis. Student's t-test and one-way ANOVA followed by Tukey's test were performed for comparisons between two groups and for comparisons of three or more groups, respectively. P<0.05 was considered statistically significant.

## Results

### MARCH6 is overexpressed in the majority of PTCs

**Figure 1:**
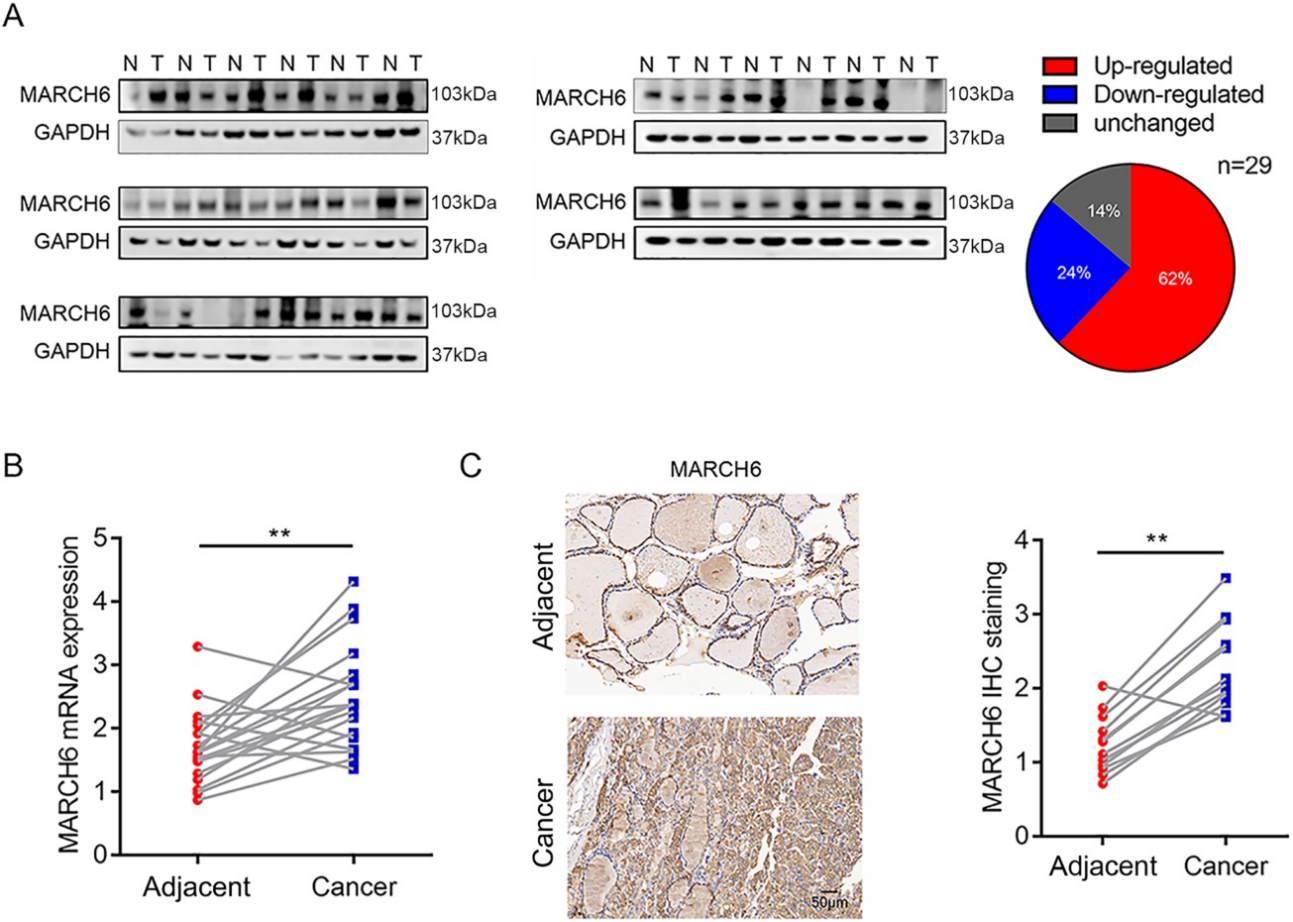
** mRNA and protein abundance of MARCH6 is increased in PTCs. (A)** Western blot analysis of MARCH6 in 29 pairs of PTCs and normal tissues showed that 62% of PTCs had upregulated MARCH6, 24% of PTCs had downregulated MARCH6, and 14% of PTCs had unchanged MARCH6. **(B)** qRT-PCR analysis of MARCH6 in 19 pairs of PTCs and normal tissues, **p<0.01. **(C)** IHC staining of MARCH6 in 12 pairs of PTCs and normal tissues. Scale bars: 50 µm, **p<0.01.

First, we collected 29 pairs of PTC tissues and adjacent noncancerous tissues (control tissues) and subjected them to immunoblotting analysis of MARCH6. A total of 18 PTCs had higher MARCH6 expression than the control tissues. Four PTCs showed comparable MARCH6 abundance, and 7 PTCs had downregulated MARCH6 expression compared with the control tissues (Figure 1A). Then, the mRNA level of MARCH6 was measured using qRT-PCR assay in paired PTCs and control tissues. We observed that MARCH6 was upregulated in PTCs (Figure 1B). Accordingly, the IHC staining results showed that MARCH6 was more abundant in PTCs (Figure 1C). These results suggest that MARCH6 may be a potential oncogene in PTCs.

### MARCH6 promotes thyroid cancer cell growth

**Figure 2:**
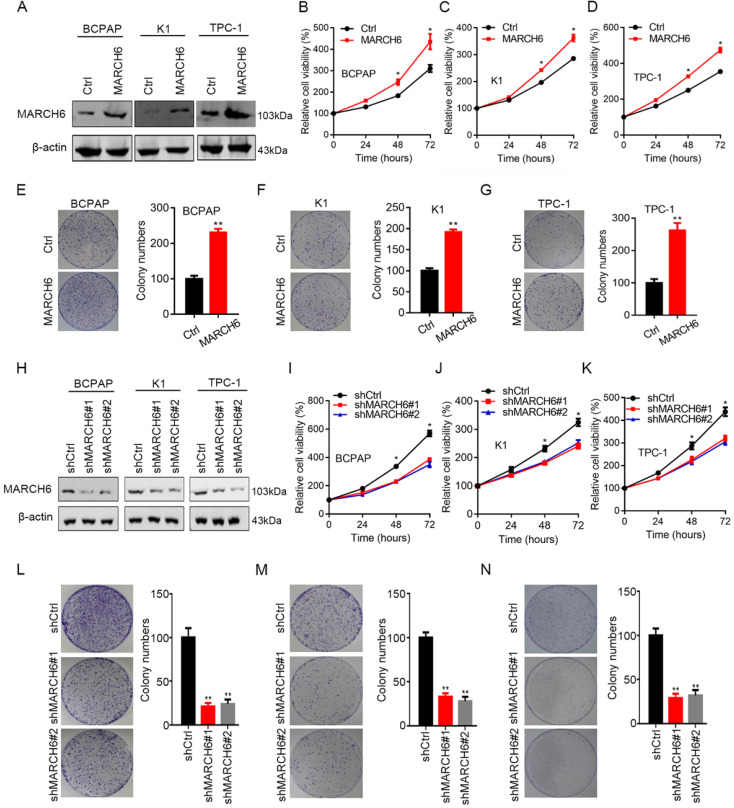
** MARCH6 contributes to thyroid cancer cell proliferation. (A)** Ctrl and MARCH6 overexpressing thyroid cancer cells were subjected to immunoblotting assays. (B-D) CCK-8 determined cell proliferation in Ctrl and MARCH6 cells. (E-G) Colony formation assays were performed in Ctrl and MARCH6 overexpressing BCPAP (E), K1 (F) and TPC-1 (G) cells. (H) Western blot analysis of MARCH6 in shCtrl, shMARCH6#1 and shMARCH6#2 thyroid cancer cells. **(I-K)** Cell proliferation was detected by CCK8 assay in shCtrl, shMARCH6#1 and shMARCH6#2 BCPAP (I), K1 (J) and TPC-1 (K) cells. **(L-N)** Colony formation assays were performed in shCtrl, shMARCH6#1 and shMARCH6#2 BCPAP (L), K1 (M) and TPC-1 (N) cells. *p<0.05, **p<0.01.

To elucidate the role of MARCH6 in thyroid cancer, gain-of-function and loss-of-function experiments were conducted in thyroid cancer cells. MARCH6 was overexpressed in BCPAP, K1 and TPC-1 cells using a lentivirus, and our immunoblotting results confirmed the overexpression efficacy (Figure 2A). CCK-8 analysis of cell proliferation demonstrated that ectopic expression of MARCH6 promoted the growth and proliferation of thyroid cancer cells (Figure 2B-2D). In addition, overexpression of MARCH6 also enhanced the colony formation of thyroid cancer cells (Figure 2E-2G). To validate the oncogenic role of MARCH6, we further silenced MARCH6 in thyroid cancer cells by two shRNA sequences. The western blot results showed that MARCH6 was efficiently knocked down in BCPAP, K1 and TPC-1 cells (Figure 2H). Silencing MARCH6 resulted in suppressed proliferation and colony growth in BCPAP, K1 and TPC-1 cells (Figure 2I-2N). Thus, MARCH6 is a potential oncogene in thyroid cancer.

### MARCH6 regulates the cell cycle and apoptosis in thyroid cancer cells

**Figure 3:**
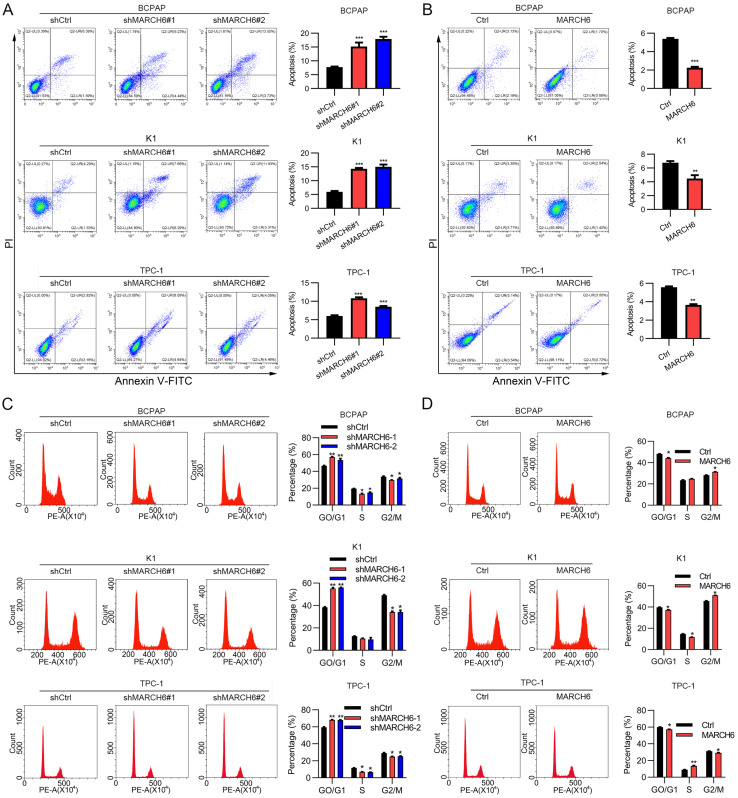
** MARCH6 suppresses apoptosis and induces cell cycle progression. (A)** Apoptosis was analyzed by PI/Annexin V-APC staining and flow cytometry in shCtrl, shMARCH6#1 and shMARCH6#2 BCPAP, K1 and TPC-1 cells, ***p<0.001. **(B)** Apoptosis was analyzed by PI/Annexin V-APC staining and flow cytometry in Ctrl and MARCH6-overexpressing BCPAP, K1 and TPC-1 cells, **p<0.01. ***p<0.001. **(C)** The cell cycle was analyzed by PI staining and flow cytometry in shCtrl, shMARCH6#1 and shMARCH6#2 BCPAP, K1 and TPC-1 cells. *p<0.05, **p<0.01. **(D)** The cell cycle was analyzed by PI staining and flow cytometry in Ctrl and MARCH6-overexpressing BCPAP, K1 and TPC-1 cells, *p<0.05.

To explore the role of MARCH6 in apoptosis, MARCH6-overexpressing and MARCH6-silenced thyroid cancer cells were stained with PI and Annexin V-APC. Then, apoptosis was analyzed by flow cytometry, and the results showed that apoptosis was induced in shMARCH6#1 and shMARCH6#2 thyroid cancer cells compared with shCtrl cells (Figure 3A). In contrast, ectopic MARCH6 expression reduced the apoptosis of BCPAP, K1 and TPC-1 cells (Figure 3B). To assess the role of MARCH6 in the cell cycle, we stained MARCH6-overexpressing and MARCH6-silenced thyroid cancer cells with PI. The cell cycle was analyzed by flow cytometry. We found that MARCH6 knockdown led to an increased G0/G1 phase and decreased G2/M phase distribution of thyroid cancer cells in the cell cycle (Figure 3C). Conversely, MARCH6 overexpression resulted in reduced G0/G1 and enhanced G2/M phase cell distribution (Figure 3D). Overall, MARCH6 suppresses apoptosis and promotes cell cycle progression in thyroid cancer cells.

### MARCH6 enhances migration in thyroid cancer cells

**Figure 4:**
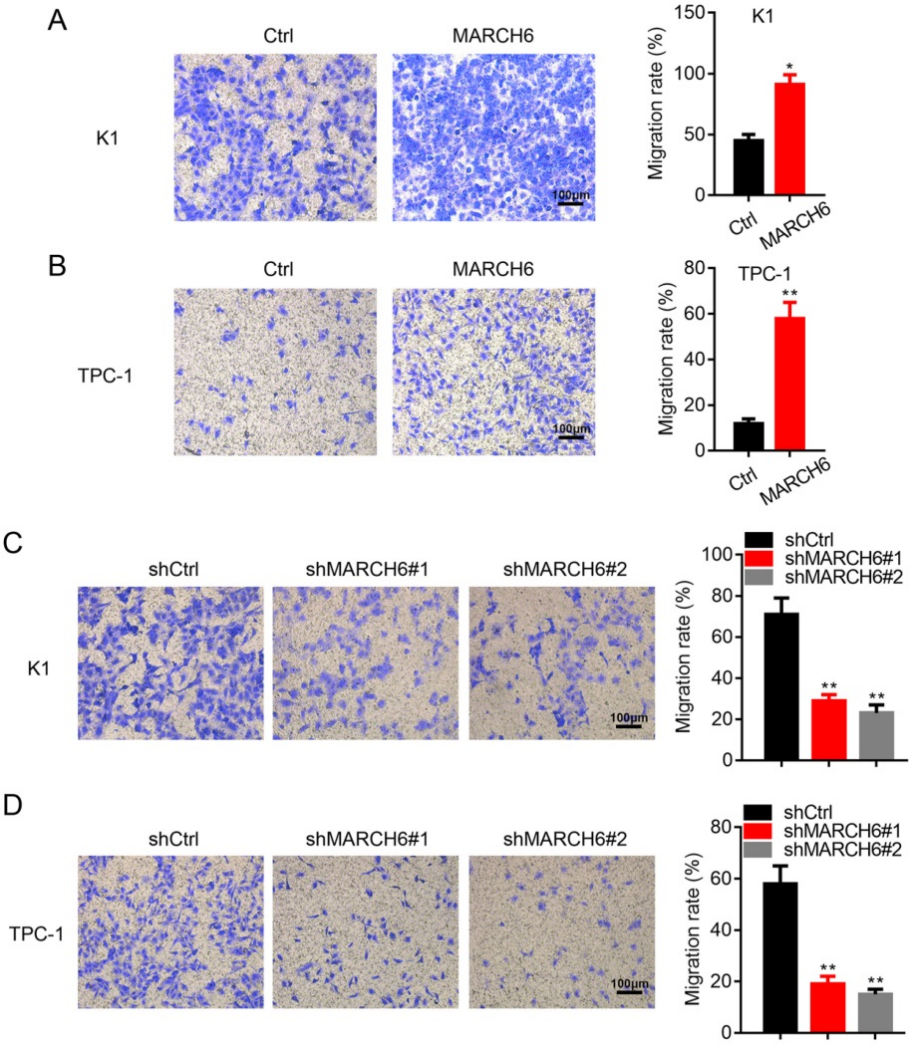
** MARCH6 promotes migration in thyroid cancer cells. (A and B)** Migration was examined by transwell assay in Ctrl and MARCH6-overexpressing cells. Scale bars: 100 µm. **(C and D)** Transwell assay determined cell migration in Ctrl and MARCH6 knockdown cells. Scale bars: 100 µm. *p<0.05, **p<0.01.

We next assessed the effect of MARCH6 on thyroid cancer cell migration. As expected, overexpression of MARCH6 enhanced the migration of K1 and TPC-1 cells (Figure 4A and 4B), while migration was significantly inhibited in shMARCH6#1 and shMARCH6#2 K1 and TPC-1 cells compared to shCtrl cells (Figure 4C and 4D). Therefore, MARCH6 promotes cell migration in thyroid cancer.

### MARCH6 promotes xenograft tumorigenesis of thyroid cancer cells

**Figure 5:**
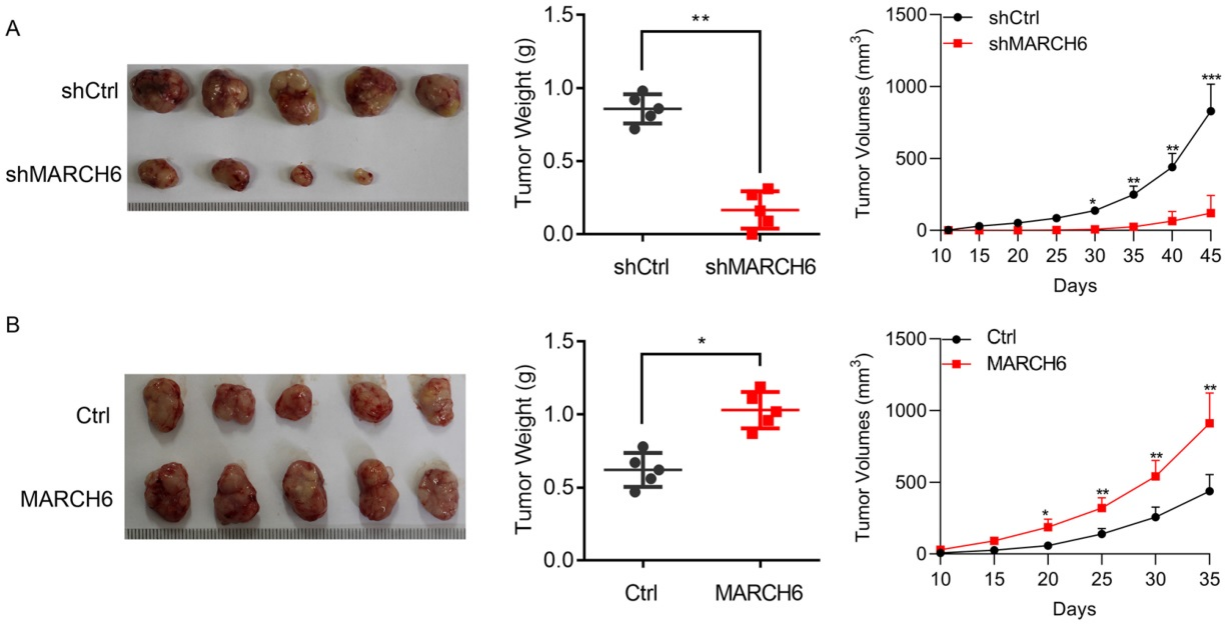
** The effect of MARCH6 knockdown and overexpression on xenograft tumorigenesis of TPC-1 cells. (A)** Equal numbers of shCtrl and shMARCH6 TPC-1 cells were subcutaneously implanted into 4-6-week-old female mice. The mice were euthanatized 45 days post implantation. Tumors were imaged and weighted. Tumor growth curve was shown. *p<0.05, **p<0.01, ***p<0.001. **(B)** Equal numbers of Ctrl and MARCH6-overexpressing TPC-1 cells were subcutaneously implanted into 4-week-old female mice. The mice were euthanatized 35 days post implantation. Tumors were imaged and weighted. Tumor growth curve was shown. *p<0.05, **p<0.01.

To elucidate the role of MARCH6 in thyroid cancer *in vivo*, lentivirus-mediated MARCH6 loss-of-function and gain-of-function experiments were performed in TPC-1 cells. Equal numbers of cells were subcutaneously implanted into 4-6-week-old female nude mice. After 35 (for Ctrl and MARCH6 overexpression group) or 45 days (for shCtrl and shMARCH6 group), the mice were euthanized, and tumors were collected for imaging and weighing. The results showed that MARCH6 knockdown suppressed the tumor growth of TCP-1 cells (Figure 5A). In contrast, ectopic MARCH6 expression accelerated the tumorigenesis of TPC-1 cells (Figure 5B). Collectively, these results indicate that MARCH6 expression is critical for thyroid cancer growth *in vivo*.

### MARCH6 interacts with and destabilizes DHX9 in thyroid cancer cells

**Figure 6:**
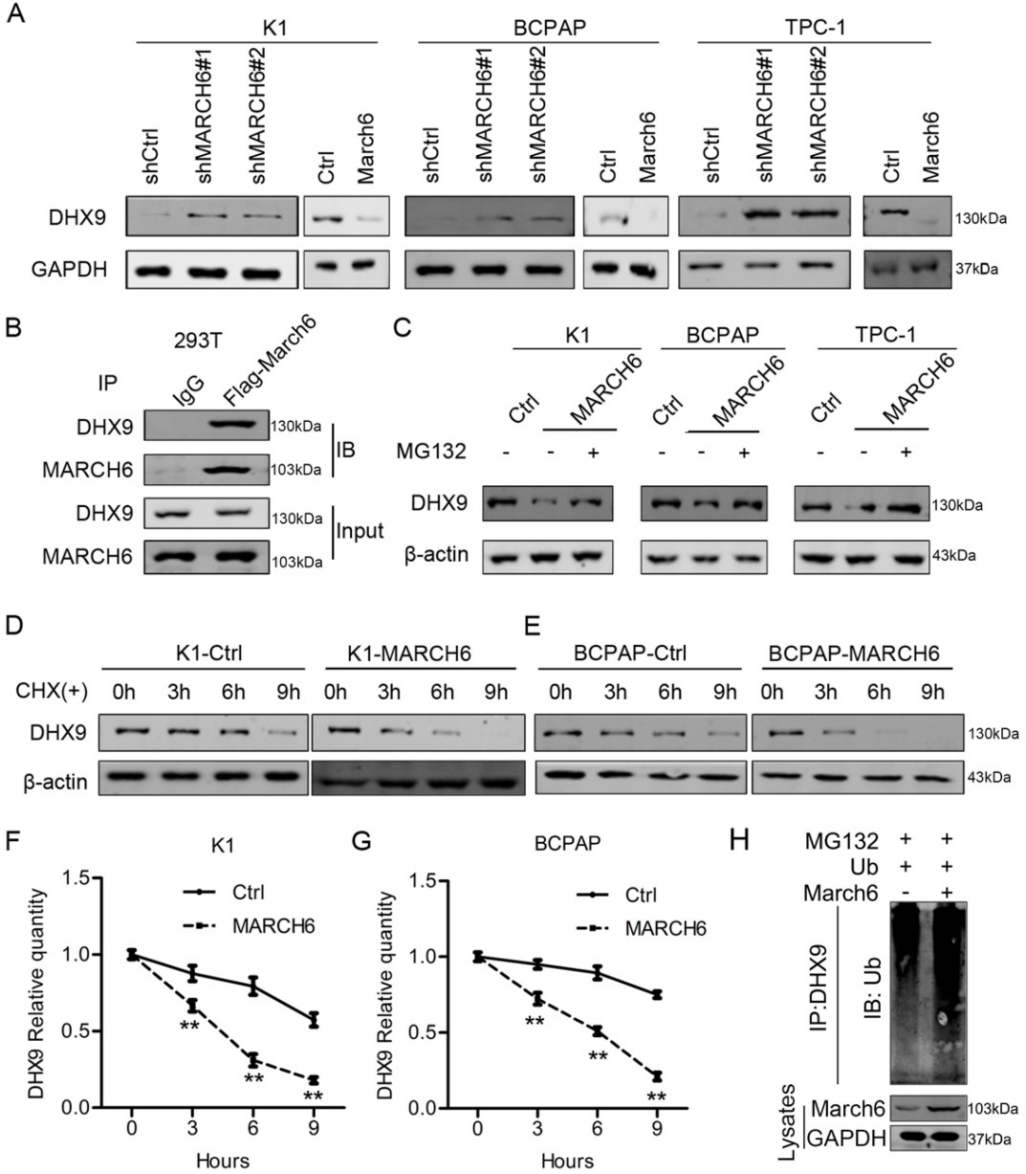
** MARCH6 downregulates DHX9 via ubiquitination. (A)** Immunoblotting analysis of DHX9 in MARCH6-knockdown and MARCH6-overexpressing thyroid cancer cells. **(B)** MARCH6-Flag was overexpressed in HEK293 cells. Cell lysates were subjected to immunoprecipitation with IgG and Flag antibodies and immunoblotting with MARCH6 and DHX9. **(C)** Ctrl and MARCH6-overexpressing cells were treated with MG132. Immunoblotting was used to detect DHX9. **(D-G)** Ctrl and MARCH6-overexpressing cells were treated with cycloheximide. Immunoblotting was used to detect DHX9, **p<0.01. **(H)** Ubiquitin was simultaneously overexpressed in Ctrl and MARCH6 overexpressing TPC-1 cells. The cells were then subjected to IP with DHX9 antibody. Ubiquitionation levels were analyzed by immunoblotting assay.

As an E3 ubiquitin ligase, MARCH6 may interact with and promote the degradation of downstream proteins. To identify the molecule responsible for the oncogenic role of MARCH6 in thyroid cancer, immunoblotting assays were performed in K1, TPC-1 and BCPAP cells with low and high MARCH6 expression. We found that MARCH6 knockdown upregulated DHX9 in these cells, while ectopic MARCH6 expression downregulated DHX9 (Figure 6A). In addition, reciprocal immunoprecipitation results showed that MARCH6 interacts with DHX9 (Figure 6B). To examine the effect of MARCH6 on the ubiquitination of DHX9, Ctrl and MARCH6-overexpressing cells were treated with MG132 and subjected to immunoblotting analysis of DHX9. The results showed that MARCH6 obviously increased the ubiquitination of DHX9 (Figure 6C). Subsequently, Ctrl and MARCH6-overexpressing cells were treated with cycloheximide, a protein synthesis inhibitor, and subjected to immunoblotting analysis of DHX9. We observed that DHX9 degradation was faster in MARCH6-overexpressing cells than in Ctrl cells (Figure 6D-6G). Lastly, we showed that MARCH6 overexpression enhanced the ubiquitination levels of DHX9 in TPC-1 cells (Figure 6H). These results indicate that MARCH6 destabilizes DHX9 by interacting with it and enhancing its ubiquitination.

### DHX9 is a tumor suppressor in thyroid cancer

**Figure 7:**
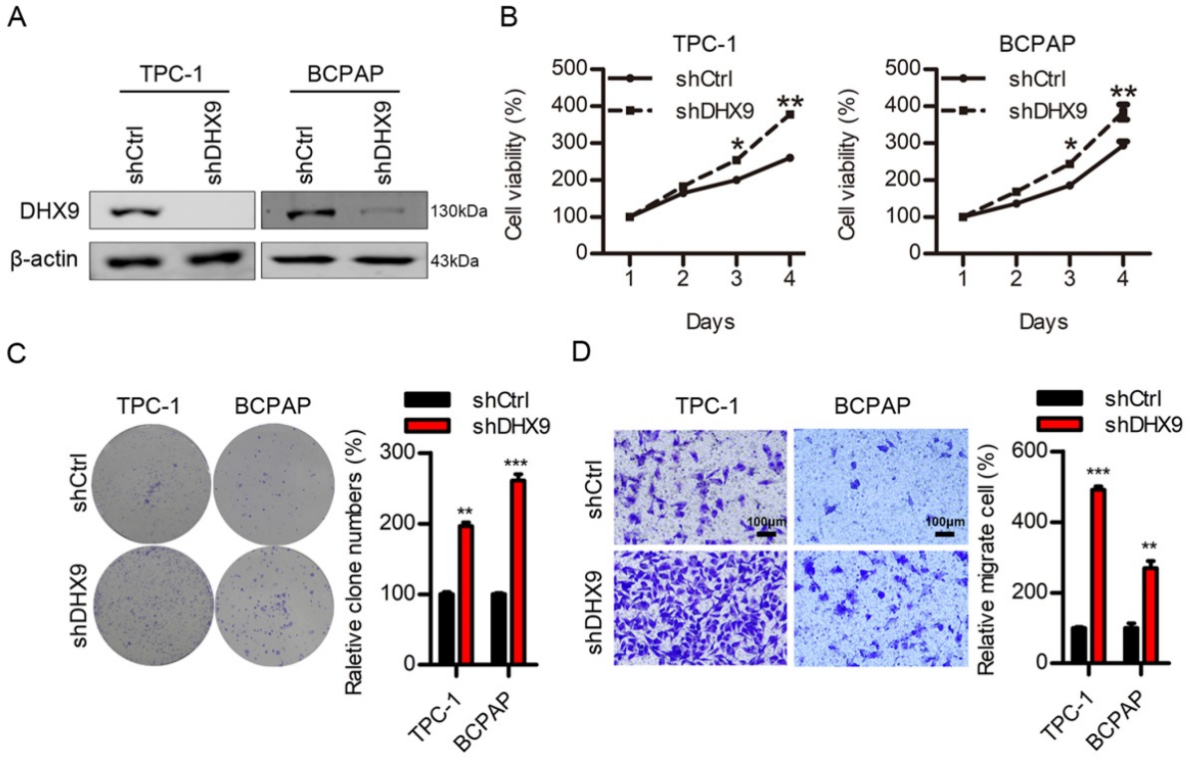
** DHX9 knockdown promotes cell proliferation and migration. (A)** Immunoblotting analysis of DHX9 in shCtrl and shDHX9 TPC-1 and BCPAP cells. **(B)** Cell proliferation was detected in shCtrl and shDHX9 TPC-1 and BCPAP cells. *p<0.05. **p<0.01. **(C)** Colony formation was assessed in shCtrl and shDHX9 TPC-1 and BCPAP cells. **p<0.01, ***p<0.001. **(D)** Migration was examined by Transwell assays in shCtrl and shDHX9 TPC-1 and BCPAP cells. Scale bars: 100 µm. **p<0.01, ***p<0.001.

The above results demonstrated that MARCH6 promoted thyroid cancer development and downregulated DHX9. To determine the role of DHX9 in thyroid cancer, we used lentivirus to knock down DHX9. The immunoblotting results showed that DHX9 was efficiently silenced in these cells (Figure 7A). Loss of function of DHX9 enhanced the proliferation, colony formation and migration of thyroid cancer cells (Figure 7B-7D). Therefore, DHX9 is likely a tumor suppressor in thyroid cancer.

### MARCH6 activates AKT/mTOR signaling through downregulation of DHX9

**Figure 8:**
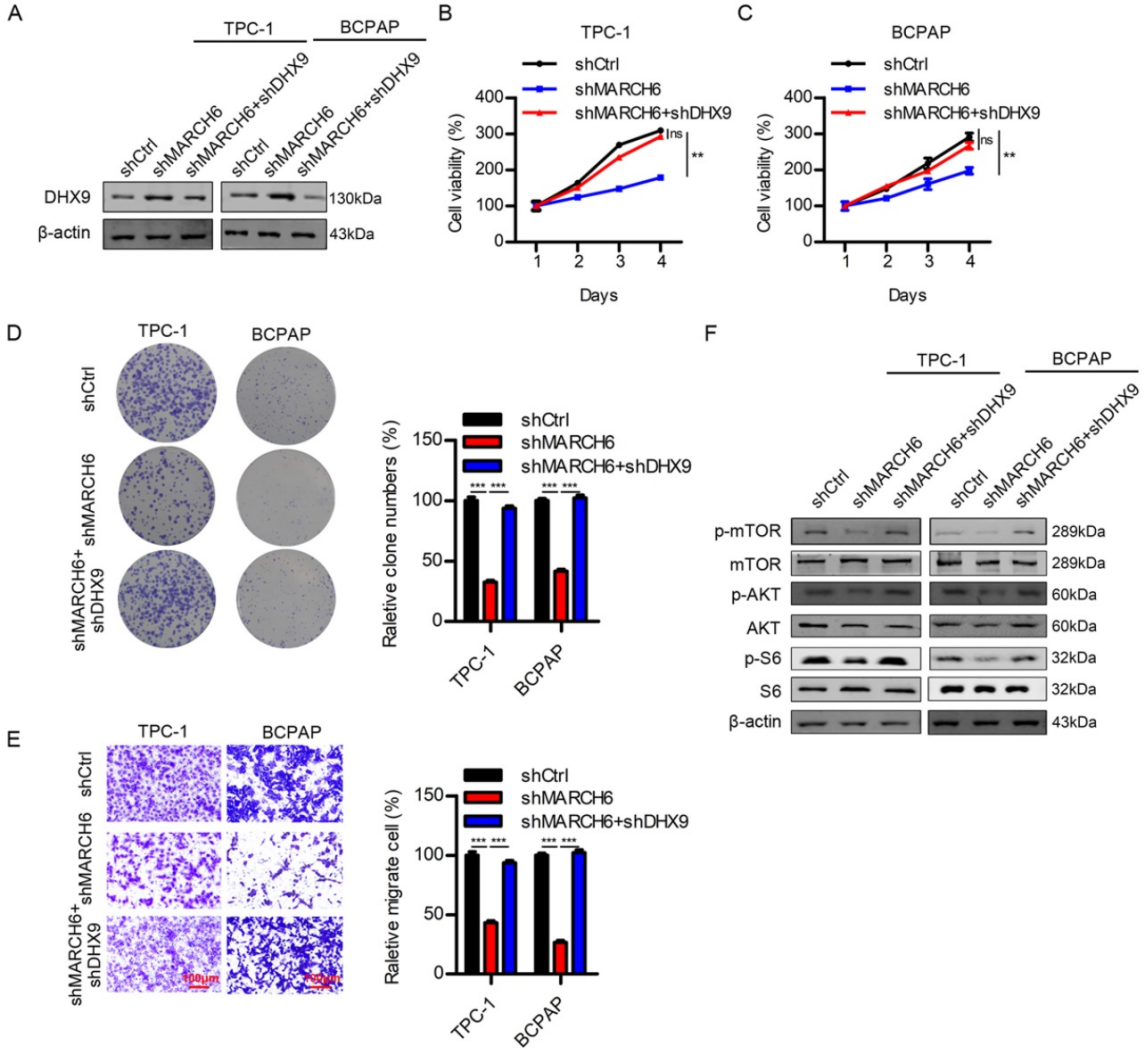
** MARCH6 regulates the AKT/mTOR signaling pathway via DHX9. (A)** shCtrl, shMARCH6 and shMARCH6+shDHX9 TPC-1 and BCPAP cells were subjected to immunoblotting analysis of DHX9. **(B and C)** Cell proliferation was detected by the CCK-8 assay in the cells shown in A. ns, no significance, **p<0.01. **(D)** The cells were subjected to colony formation analysis, ***p<0.001. **(E)** The cells were subjected to Transwell migration assays. Scale bars: 100 µm. ***p<0.001. **(F)** The cells were subjected to immunoblotting with the indicated antibodies.

Since MARCH6 silencing upregulated DHX9 in thyroid cancer cells, we interfered with DHX9 expression in cells with downregulated MARCH6. The immunoblotting results confirmed the efficacy of DHX9 knockdown (Figure 8A). As expected, when MARCH6 knockdown suppressed proliferation, colony formation and migration, further silencing DHX9 reversed these phenotypes in thyroid cancer cells (Figure 8B-8E). Interestingly, MARCH6 downregulation inhibited the phosphorylation of AKT, mTOR and S6, which could be reversed by DHX9 silencing (Figure 8F). These results suggest that MARCH6 activates the AKT/mTOR signaling pathway by repressing the expression of DHX9.

## Discussion

The overall 10-year survival rate of thyroid cancer patients is relatively high following conventional treatments, but a small number of patients still suffer from lymph node metastasis, tumor recurrence and drug resistance 19. Recent studies have revealed that the protein ubiquitin pathway is a crucial player in the progression of thyroid cancer 20-22. MARCH proteins, which belong to E3 ubiquitin ligase family, are essential post-transcription regulators that promote protein degradation by increasing ubiquitin. As a member of the ubiquitin pathway, MARCH6 has been reported to act either as a tumor suppressor or an oncogene in solid tumors 13. For instance, MARCH6 was upregulated and promoted proliferation in breast cancer cells 12. In prostate cancer, MARCH6 expression was positively correlated with androgen receptor expression 23. However, whether MARCH6 has potentially important roles in the occurrence and progression of thyroid cancer still needs to be fully clarified. In this study, we observed a high expression of MARCH6 in PTC tissues. MARCH6 promoted cell growth of thyroid cancer cells *in vitro* and *in vivo*. MARCH6 also promoted cell cycle progression and suppressed apoptosis in thyroid cancer cells. In addition, MARCH6 overexpression contributed to the migration ability of TPC-1 and K1 cell lines which are originated from papillary carcinoma. The present findings indicated that MARCH6 has oncogenic activity in thyroid cancer.

Although the high expression of MARCH6 was strongly associated with thyroid cancer cell proliferation, migration, cell cycle progression and cell apoptosis, the underlying mechanisms involved are still poorly understood. Indeed, the E3 ubiquitin ligase MARCH6 is required for selective substrate degradation and protein stability by stimulating ubiquitination 8, 24. To explore the molecular mechanism by which MARCH6 functions in thyroid cancer, we performed a co-IP assay to identify the potential protein that could interact with MARCH6. Consequently, we found that MARCH6 knockdown upregulated DHX9, while its overexpression downregulated DHX9. MARCH6 would interact with DHX9 and destabilize DHX9 in thyroid cancer cells. Therefore, DHX9 is the substrate of MARCH6 in thyroid cancer.

DHX9, a member of the DExD/H-box family of helicases, is highly expressed in various malignant tumors and has been confirmed as a homologue of the Drosophila Maleless (MLE) protein 25. DHX9 appears to play pivotal roles in multiple cellular processes, including the regulation of DNA replication, transcription, translation, RNA processing and maintenance of genomic stability and is thus associated with several human diseases 26, 27. A previous study indicated that DHX9 inhibited the EMT process in human lung cancer cells by regulating STAT3 16. DHX9 could also interact with the 3' untranslated region to repress the expression of CDK6, a well-known oncogene 28. However, DHX9 seems to be an oncogene in some cancers. For example, DHX9 was highly expressed in cervical cancer and promoted cell motility and angiogenesis 29. In addition, speckle-type POZ protein functioned as a tumor suppressor in choriocarcinoma through downregulating DHX9 at ubiquitination manner^30^. These results indicate that DHX9 may exhibit distinct functions in the development of different cancers. Besides, the upstream regulators of DHX9 remain to identify. Since DHX9 was targeted by MARCH6 in our study, the function of DHX9 in development of PTC should be determined. As our results showed, silencing DHX9 inhibited cell proliferation, migration and cell cycle progression and induced cell apoptosis in thyroid cancer cells, indicating that DHX9 acted as a tumor suppressor in thyroid cancer.

The AKT/mTOR signaling pathway is frequently activated in a wide variety of cancers. This signaling pathway plays important roles in regulating cell proliferation, migration, cell apoptosis and cell survival 31-33. Previous studies have suggested that activation of the AKT/mTOR pathway induces thyroid cancer cell proliferation and migration 34, 35. However, the upstream inducer and regulator of AKT/mTOR signaling pathway remain to be determined in thyroid cancer. The association between DHX9 and AKT/mTOR is also unclear. In this study, we showed that MARCH6 MARCH6 would activate AKT/mTOR signaling pathway, which may contribute to thyroid cancer cell growth and migration. Interestingly, the activation of AKT/mTOR in MARCH6 overexpressed cells depended on the downregulation of DHX9. Taken together, our results confirm that MARCH6 promotes the growth and migration of thyroid cancer cells via activation of the AKT/mTOR pathway.

In summary, MARCH6 significantly promotes thyroid cancer growth and migration by interacting with DHX9 and activating the AKT/mTOR pathway. These findings prove that MARCH6 might serve as a potential novel target for the early diagnosis of thyroid cancer.

## Abbreviations

MARCH: Membrane-associated ring-CH-type finger
PTCs: papillary thyroid cancers
TC: Thyroid cancer
DHX9: DExH-box RNA helicase 9
DSB: double-strand break
CCK-8: Cell Counting Kit-8
PI: propidium iodide
MLE: Maleless
